# Culinary Education Programs for Children in Low-Income Households: A Scoping Review

**DOI:** 10.3390/children7050047

**Published:** 2020-05-13

**Authors:** Priscilla P. Li, Guisela Mackey, Chishinga Callender, Jayna M. Dave, Norma Olvera, Shana Alford, Debbe Thompson

**Affiliations:** 1 USDA/ARS Children’s Nutrition Research Center, Department of Pediatrics, Baylor College of Medicine, 1100 Bates Street Houston, TX 77030, USA; priscipli02@gmail.com (P.P.L.); Guisela.Mackey@bcm.edu (G.M.); Chishinga.Callender@bcm.edu (C.C.); jmdave@bcm.edu (J.M.D.); 2 Psychological, Health, and Learning Sciences Department, University of Houston, 3657 Cullen Boulevard Room 491, Houston, TX 77204, USA; nolvera@central.uh.edu; 3 Common Threads, 222 W. Merchandise Mart Plaza, Suite 1212, Chicago, IL 60654, USA; salford@commonthreads.org

**Keywords:** culinary education, elementary aged children, low-income

## Abstract

Child obesity in the United States is at an all-time high, particularly among underserved populations. Home-cooked meals are associated with lower rates of obesity. Helping children develop culinary skills has been associated with improved nutrition. The purpose of this study is to report results from a scoping review of culinary education interventions with children from low-income families. Three databases and hand searches of relevant articles were examined. Retained articles met inclusionary criteria. The Preferred Reporting Items for Systematic Reviews and Meta-Analyses (PRISMA) guidelines were followed, as appropriate. A data extraction template was developed. Data were independently extracted and verified. Only nine out of 370 articles met the inclusionary criteria and were included in the review. Most interventions were school-based, used a quasi-experimental design, and recruited minority children. Children-only was the primary intervention focus. Primary outcomes were mostly psychosocial from child self-report. Most interventions focused on children only and were guided by Social Cognitive Theory. Most reported stakeholder involvement; however, type and degree varied. All had an in-person component; only one used technology. Few reported training program leaders. Culinary education programs for children from low-income families could benefit from a broader theoretical grounding, program leader training, and greater parental involvement.

## 1. Introduction

Child obesity in the United States is at an all-time high. Among 2–19-year-olds, 35.1% are overweight and, of these, 18.5% are obese [1]. However, the risk is not equally distributed, with alarming disparities observed based on race/ethnicity [2] and household income [3]. Finding effective ways to overcome these disparities in obesity risk is a national health priority [4].

Although not a prerequisite for a healthy diet [5], consuming home-cooked meals is associated with lower rates of obesity [6] and better diet quality in both adults and children [7,8,9,10,11]. Alternatively, meals prepared outside the home are associated with poorer food choices [12,13,14], greater energy intake [15], and higher body mass index [16]. Time spent on home food preparation has decreased [17], with fewer families preparing and consuming home-cooked meals [18]. People are purchasing foods, such as fast foods, and consuming them at home [15]. Home food delivery is popular [19], with reasons ranging from not wanting to cook to saving time [20].

Changing times have likely contributed to this shift in home food preparation and consumption. More women in the workforce [21], single parent households [22], demanding schedules [11], and long commutes [23] have reduced time available for meal planning, shopping, and preparation. Personal finances, negative cooking experiences, desire for effortless meals, and family preferences have also been cited as obstacles to home cooking [24].

Contrary to general trends described above, a report on cooking habits of low-income families revealed that most prepared meals at home, particularly dinner [25]. Although families expressed a desire to serve healthy meals, they found it difficult to do so. Price and beliefs (e.g., frozen foods are less healthy) were identified as barriers. Research with food pantry clients support these findings [26]. The built environment has also been shown to influence access to healthy, affordable foods. Low-income neighborhoods are more likely to have higher concentrations of less healthy food outlets, such as fast food restaurants and convenience stores [27] and fewer grocery stores [28]. These findings suggest that interventions for low-income families should be tailored to their specific needs, beliefs, and circumstances.

Children have a substantial influence on the home food environment [29]. Learning culinary skills at an early age increases frequency of meal preparation at home, and thus can lead to less reliance on take-out or outside foods [30]. Positive associations have been reported among youth involvement in home meal preparation and improvement in vegetable preference, self-efficacy for cooking and choosing healthy foods, fruit and vegetable consumption, and overall dietary quality [31]. Helping with home meal preparation is a youth behavior that is realistically modifiable and may substantially influence overall dietary quality [31].

Previous reviews of culinary education programs for school-aged children [32,33] have not specifically focused on children from low-income families. Given that families living in low-income households are likely to face different nutrition-related challenges than their affluent counterparts, this is an important gap in the literature. Therefore, the purpose of this review is to examine culinary education interventions evaluated for children and/or their families within low-income households. Scoping reviews are conducted to identify research gaps [34] and provide suggestions for future research.

## 2. Methods

This scoping review provides an examination of interventions for children and/or their families in low-income households that included a focus on culinary skills (i.e., cooking skills). The research question for this review was: *what are the characteristics of culinary education interventions for children and/or their families living in low-income households?* Two particular interests for this review were stakeholder involvement during program development and adaptations made to address the needs of low-income children and/or their families.

### 2.1. Data Sources

The guidelines for Preferred Reporting Items for Systematic Reviews and Meta-Analyses (PRISMA) were followed as appropriate [35].

Databases searched included PubMed, Cumulative Index of Nursing and Allied Health Literature (CINAHL), and PsychInfo. Articles published between 1990 and 2017 were included in the review. Most of the articles were found using a Boolean search that used search terms based on inclusionary and exclusionary criteria. Search terms included: “cooking classes”, “parents”, “children”, “culinary skills”, “nutrition”, “intervention”, and “underserved”. The remaining articles were found using a hand search of articles included in the review after searching the three databases.

### 2.2. Study Selection

Inclusionary criteria included intervention studies that conducted cooking and culinary skills classes, parents and/or school-age children (5–18-year-olds) and reported psychosocial and/or behavioral outcomes. Exclusionary criteria included conference abstracts, review articles, programs for solely college students or adults (that did not pertain to eating habits of children), non-interventional studies (descriptive, qualitative, or cross-sectional), and studies that did not have a focus on a low-income population.

**Figure 1:**
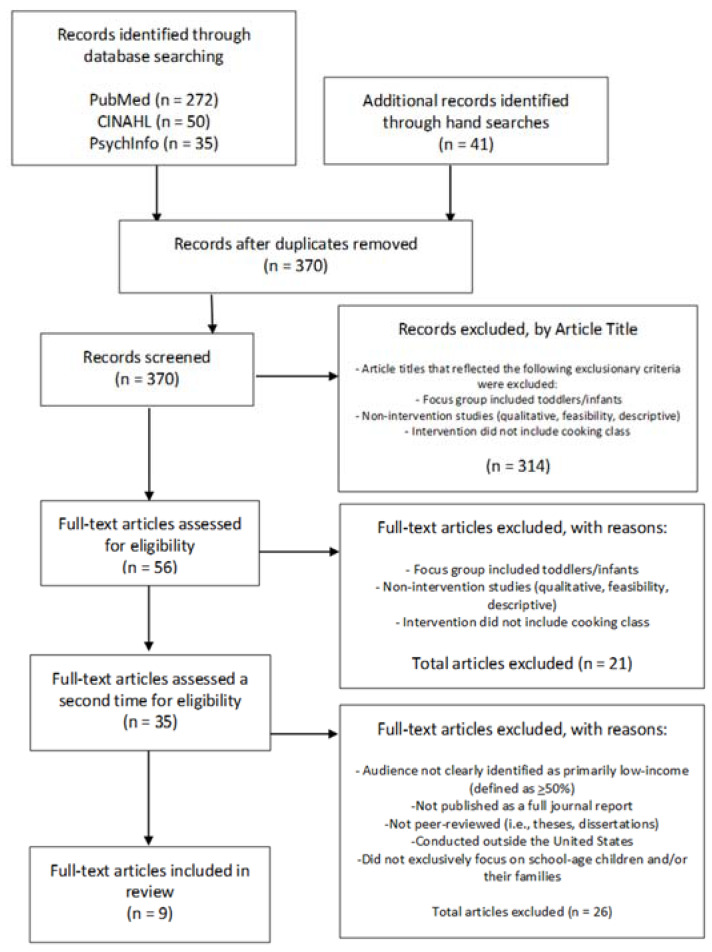
Consort Diagram.

A total of 370 articles were screened using the inclusionary and exclusionary criteria outlined above. After screening articles by title and assessing them by reading the articles, 35 full-text articles were identified that met initial inclusionary criteria. A deeper review of articles reporting interventions conducted with children and/or their families living in low-income areas were further examined to identify stakeholder involvement, adaptations/tailoring for low-income families, and program characteristics (i.e., leader training). Articles not clearly identifying the audience was primarily low-income (defined as ≥50%), not published as a full journal report, not peer-reviewed (i.e., theses, dissertations), conducted outside the United States, and/or did not exclusively focus on school-age children and/or their families were excluded from further review. Nine studies met the review criteria and were included in the focused review reported here (Figure 1).

### 2.3. Data Extraction

Data from the initial search were independently extracted by two authors; results were compared, and differences reconciled (PPL, DT). The articles meeting the second set of inclusionary criteria were further screened by two independent extractors (GM, DT) to identify characteristics of the studies (Table 1), designs (Table 2), and interventions (Table 3). A third extractor (JD) reviewed and confirmed tables.

## 3. Results

Nine studies met the criteria. Most used a quasi-experimental design and collected data at baseline and post-assessment; only two studies were randomized controlled trials. Among all studies, one study had multiple assessment points and one conducted post-intervention focus groups. All studies recruited children; however, two also recruited parents. Sample size ranged from 89–1204 participants. Eight studies recruited participants exclusively from schools, including after-school programs. Only one study recruited participants from subsidized housing complexes, churches, and community centers in addition to schools. Of the 9 studies, 7 recruited from schools with a majority of students eligible to receive free/reduced priced lunches; of the remaining two, one recruited from a school located in a low-income school district, and in the other study, families had to qualify for public assistance to be eligible to participate in the study. Six studies provided family-level socioeconomic status (SES) data; in these studies, nearly all participants qualified for free/reduced price lunch, and one reported that most families who participated had low or very low food security. In all but one study, most participants were of from an ethnic minority group (Black/African American, Hispanic/Latino). All studies were conducted in the United States: four were in Western region of the country, one in the Southwestern region, and two each in the Midwestern and Northeastern regions of the country. All studies collected data from children; three also collected data from parents. The primary method of data collection was self-report survey; however, one study conducted visual plate waste inspections, and one collected anthropometric data and offered an optional fasting blood sample. One conducted post-intervention focus groups with parents. All studies reported positive outcomes in psychosocial variables (e.g., preference, self-efficacy, etc.) (Table 1).

Five studies reported using a theoretical framework to design the study. The most common theoretical framework was Social Cognitive Theory (SCT); two studies also used Self Determination Theory (SDT) in addition to SCT. Six studies reported involving stakeholders at varying levels during intervention design. All studies reported adapting the intervention for low-income families, although the type of adaptation varied greatly (Table 2).

Interventions included a variety of components. All studies involved an in-person activity such as cooking demonstrations, food preparation, nutrition lessons, tasting sessions, and gardening activities. One study also included a virtual gardening game played on a tablet as part of the intervention. Another study provided a grocery store tour. Support materials (e.g., toolkit) or food were provided to families in three studies. The intervention focus was the child in seven studies and both child and parent in two studies. Parent involvement ranged from none to substantial. Session frequency and duration were variable, ranging from a single 20-min session per month to an immersive school-wide program lasting a school year. A variety of individuals, including classroom teachers, nutrition or food educators, chefs, and volunteers, led the programs. Only four studies mentioned training individuals to lead the intervention. Most studies were conducted at school (e.g., classroom, cafeteria, school garden, after-school program); others were conducted at host sites in the community (Table 3).

## 4. Discussion

This review identified nine studies designed to enhance culinary skills in children and/or their families living in low-income households within underserved communities. All but two of the studies were quasi-experimental, suggesting the results should be viewed with caution because of concerns related to internal validity, such as the potential for confounding and regression to the mean [45]. Given that most of the studies were conducted in a school setting where it would be difficult to randomize students to condition, future research is needed to examine ways in which to enhance the robustness of studies using a quasi-experimental design [46].

Although the focus of this review was on children and/or their parents within low-income households, a key finding was that most participants were Hispanic or Black/African American. This finding is not surprising, given the well-documented racial and ethnic disparities in income seen in the United States [47]. However, this suggests that culinary education interventions for low-income children should also consider race/ethnicity when designing the intervention. Interventions that reflect a deep cultural sensitivity and awareness of cultural norms and values in an effort to increase perceived personal relevance, usefulness, and intervention uptake is vital [48,49]. The studies included in this review reported some degree of cultural adaptation; however, the descriptions were relatively sparse. Future research should be more explicit in the steps taken to ensure cultural relevance.

Behavioral theory guided five of the identified interventions. The most commonly cited theory was SCT [50], a theory often used to guide interventions focused on dietary change [51,52]. SDT [53], a theory focused on enhancing autonomous (i.e., self-directed) motivation was also used by two of the intervention studies. Given that motivation is an important component of sustained behavior change [53] and its success at explaining behaviors related to diet and obesity such as physical activity [54], future research should investigate additional ways to design culinary education programs guided by SDT. Four of the interventions did not identify a theoretical framework. This is concerning because theory codifies what is known about a particular behavior and provides a framework for predicting and explaining behavior [55]. Therefore, it is a necessary ingredient of behavior change interventions [55]. Of the studies reporting a theoretical grounding, few described how theory guided intervention development and/or used it to explain the intervention results. This is not uncommon in behavioral research, and there have been calls to more explicitly describe how theory was applied in the design of an intervention [52,56]. Future research should investigate which theory or combination of theories is most effective at promoting culinary skills to low-income children.

Most of the studies reported that stakeholders were involved in intervention development; however, the type and degree of involvement, and who was defined as a stakeholder, varied greatly. Stakeholder involvement (i.e., the individuals, groups, or organizations affected by the research [57]) is an important aspect of intervention development [58] with promising implications for the design of effective interventions [59]. Future research should investigate ways in which to systematically engage stakeholders throughout the design process, and evaluate the association between stakeholder involvement (i.e., type, extent) and intervention effectiveness. This will contribute to the design of more effective interventions.

The child alone was the primary intervention focus in most of the interventions. Because parents are gatekeepers of the home environment [60], it would be advantageous to include parents in culinary education interventions for children. Therefore, future research should investigate ways to design culinary education programs that include both children and parents.

A variety of components was included in the interventions. All studies involved in-person activities, which is a common delivery mode for dietary interventions [52]. Given the popularity of videogames [61] and the broad ownership of devices on which games can be played [62,63], it is interesting to note that only one of the interventions included a digital component. Videogames have been found to be effective at modifying the dietary intake of children [64]. Technology-based interventions may be particularly salient in school-based culinary education programs; students report using mobile technology for schoolwork, and some schools provide students access to tablets and/or computers in the school environment [65]. Therefore, future research should identify ways in which to use technology to develop culinary education programs for low-income children and/or their families.

Program leaders varied from teachers to registered dietitians and chefs. However, it was somewhat surprising that few interventions mentioned training program leaders to deliver the intervention. Training is likely linked to fidelity, or the degree to which an intervention is delivered as intended [66]. Fidelity has been identified as a determinant of intervention efficacy [52]; thus, identifying ways to enhance fidelity is an important aspect of intervention delivery. It is possible that the type of program leader (e.g., registered dietitian vs volunteer) will influence the form and degree of training needed. Future research should investigate this issue as well as the relationships between program leader training, fidelity, and program effectiveness.

Dose is an important concept in behavioral interventions and represents the “amount” of an intervention intended and received [66]. Intervention dose in the studies included in this review varied from several sessions to an entire school year. Although there have been attempts to identify the ideal dose for behavioral interventions targeting children, no consensus has been reached [67]. Future research is necessary to improve general understanding of dose in culinary education programs, designed for low-income children.

Finally, all studies reported positive outcomes. However, most used self-report measures; only two reported objectively assessed outcomes (e.g., visual plate waste inspections; measured anthropometrics; optional blood work). This is a concern, given the known reporting bias often associated with self-report [68]. Furthermore, only one study included post-intervention focus groups. This is a missed opportunity to understand what it was like to participate in the intervention from the participant’s perspective and obtain suggestions for needed modifications. Finally, most studies assessed psychosocial outcomes rather than changes in behavior (e.g., home cooking frequency, nutritional intake). Although psychosocial outcomes are thought to be mediators of behavior [69], it would have been preferable to report intervention effects on behavior. Future research could make important contributions to the literature by reporting behavioral outcomes using objective measures when possible. Post-intervention qualitative research is needed to understand the “experience” of participating in the intervention from the perspective of families, which could ultimately guide the design of more effective and sustainable interventions that reflect the needs and interests of families [70].

As with most research, there are limitations. We limited the review to papers in peer-reviewed journals that were published in English. There may have been unpublished studies or studies conducted in other countries or reported in other languages that examined culinary interventions for low-income children and/or their families. Furthermore, the review exclusively examined studies conducted in the United States, thus limiting its generalizability. Finally, most of the studies included in the review were quasi-experimental, limiting reasonable conclusions regarding causality.

## 5. Conclusions

Culinary education for children may provide an optimal avenue for enhancing frequency of home-cooked meals and overall quality of foods consumed during childhood, and potentially in adulthood. Developing these skills may also lead to improved and sustained dietary behaviors and patterns and reduced risk of diet-related chronic disease, including obesity. Additional research is needed to enhance the design of effective interventions that achieve goals of culinary education for children and their families, especially those faced with challenges such as lower income.

## 6. Implications for Research and Practice

These findings suggest that greater emphasis needs to be placed on finding effective ways to promote culinary skills to children from low-income families in appealing, culturally appropriate ways. Greater emphasis needs to be placed on developing programs for parent-child dyads, involving stakeholders in program development, using theory to guide intervention content and development, and training program leaders to ensure programs are delivered as intended.

## Figures and Tables

**Table 1:** Study characteristics.

Author (Year)	Study Name	Research Design	Data Collection Schedule	Recruitment Focus	Sample Size	Income/SES Criterion	Participant Race/Ethnicity	Recruitment Location	Geographic Location	Data Collection Method	Results
Bell et al. [36] (2018)	Virtual Sprouts	two group design; quasi-experimental; pilot intervention	pre+post (child only)	child (predominantly minority, underserved; 3–5 grades)	180 (control = 64, intervention = 116)	public elementary charter schools in LA; participants: 92% treatment/73% control eligible to receive free lunch	Latino 9.5% tx/11.3% control; White 0% tx/1.6% control; Black 63% tx/58.1% control; Native American 0.9% tx/0% control; Mixed Race 25.9% tx/29% control; Other. 9% tx/0% control	School (n = 2)	Los Angeles, CA	survey	+ self-efficacy to eat FV; + self-efficacy to cook FV
Chen et al. [37] (2014)	Cooking up Diversity	two group design; quasi-experimental; mixed methods	pre+post (child + parent); post-intervention focus groups (parent only)	both (K-2 students)	1204 (control = 600; intervention = 604)	low-income schools where majority of students were eligible to receive free/reduced price meals program; participants: nearly 80% qualified for free/reduced price meals	Latino/Hispanic 32.4%; Hmong 9.1%; White 42.3%; Other 16.2%	School (n = 6)	Northern California	survey; focus group discussions	+ familiarity, preferences, and consumption of vegetables and increased involvement with food prep at home; + parental appreciation of new foods/recipes
Cunningham-Sabo et al. [38] (2014)	Cooking with Kids	(2 cohorts); 3 group design; quasi-experimental	pre+post (child only)	child (4th grade)	961 (completed both pre and post-survey)	schools had to have ≥50% of students eligible for free/reduced price school meals; participants: SES not provided	Hispanic 84.1%; White 10.1%; American Indian 2.8%; American Indian 2.8%; Black 1.1%; Asian 0.6%; NA 1.3%	School (n = 11)	Santa Fe, NM	survey	+FV preferences +cooking self-efficacy and attitudes in students without cooking experience (mostly males)
D’Adamo et al. [39] (2016)	Spice MyPlate	quasi-experimental; two group design	baseline, 3, 6, and 10 weeks after baseline (child only)	child	110	School—free/reduced price meal participation (School A = 75%; School B = 74%); participants—SES not provided	African American 87.3% tx/63.6% control; White 1.8% tx/12.7% control; Hispanic 0% tx/3.6% control; Asian/Pacific Islander 0% tx/3.6% control; Native American 3.6% tx/0% control; Other 1.8% tx/9.1% control	school (n = 2) (grades 9–12)	East Baltimore, MD	3-day food record, survey	Spice MyPlate intervention was feasible; + whole grains, and protein foods intake; + attitudes towards eating vegetables, whole grains, lean protein, and low-fat dairy
Davis et al. [40] (2016)	LA Sprouts	RCT	pre+post (child only)	child (3rd–5th grade)	304 (control = 137; intervention = 167)	school eligibility: ≥75% received free/reduced price lunches; participants—89% control/91% treatment eligible for free/reduced price lunch	Hispanic 88.8% control/88.6% tx; Asian 1.5% control /0.6% tx; Non-Hispanic Black 0% control/2.4% tx; Non-Hispanic White 1.5% control/1.2% tx; Other 8.2% control/7.2% tx	after-school program (n = 4)—LA’s Better Educated Students for Tomorrow	Los Angeles, CA	Questionnaire	+ Scores for identification of vegetables, and nutrition and gardening knowledge for LA Sprouts participants; + More likely for LA Sprouts participants to garden at home
Gatto et al. [41] (2017)	LA Sprouts	2 group RCT	pre+post (child only)	child (3–5 grades)	319 (control = 147; intervention = 172)	school eligibility: ≥75% received free lunch program; participants—89% control/91% treatment eligible for free/reduced price lunch	Hispanic/Latino 89% tx/88.8% control	after-school program n = 4)—LA’s Better Educated Students for Tomorrow	Los Angeles, CA	food frequency questionnaire, anthropo-metrics, optional fasting blood sample	LA Sprouts participants had greater reduction in BMI z-scores, and waist circumference; − Number of LA Sprouts participants with metabolic syndrome; + Dietary fiber intake for LA Sprouts participants; − Decreased vegetable intake for all study participants, but LA Sprouts participants had smaller decreases
Jarpe-Ratner et al. [42] (2016)	Common Threads	quasi-experimental	pre+post (child + parent)	child (grades 3–8)	271	≥80% of students eligible for free/reduced price lunch; participants—94% eligible for free/reduced price lunch	(analyzed sample) African American 44%; Hispanic 42%; White 7%; Other 7%	School (n = 18)	Chicago, IL	survey	+ FV consumption, nutrition knowledge, cooking self-efficacy, exposure to new foods, and cooking at home for students; + Family conversations about healthy foods, frequency children prepared dinner, parent perception on ability to prepare health meal, and importance parents place on family meal; sustained effect at post 2
Liquori et al. [43] (1998)	The Cookshop Program	quasi-experimental design	pre+post (child only)	child (K-6 grades)	590	schools: low-income school district; participant SES not provided	not provided for participants; however, recruited from schools that were 85% African American and 15% Hispanic	School (n = 2)	Central Harlem community of NYC	survey; visual inspection of plate waste	+ (CS) preferences, knowledge, and plate waste in both younger and older children and on behavioral intention in younger children and cooking self-efficacy in older children; + (FEL) knowledge for both age groups
Overcash et al. [44] (2018)	Cooking Matters for Families	one group; quasi-experimental	pre+post (child + parent)	both	89	family qualified for public assistance; participants—61% had low/very low food security	White 12%; Black/African American 34%; Asian/Pacific Islander/American Indian 4%; Other 41%; Mixed race 9%; Hispanic ethnicity 43%	Subsidized housing, churches, schools, and community centers (# of participating organizations not identified)	Minneapolis-St Paul, MN	survey	+ Parental cooking confidence, healthy food prep, child self-efficacy, vegetable variety and home vegetable availability

BMI, body mass index; FV, fruits and vegetables; SES, socioeconomic status; tx, treatment group.

**Table 2:** Design characteristics.

Author (Year)	Theoretical Framework (s)	Stakeholder Involvement	Adaptation for Low SES
Bell et al. [36] (2018)	Self Determination Theory, Social Cognitive Theory	formative research with stakeholders to develop the program	extension of previous nutrition/cooking/gardening program for urban Latino upper elementary aged children; formative work with stakeholders (observation, focus groups, surveys, prototyping, concept testing)
Chen et al. [37] (2014)	none described	parents, bicultural staff members who had experience providing cooking classes to Hmong/Latino adults participated in recipe development	Local, ethnic produce items were featured. Ingredients were affordable and provided to students. Equipment such as cutting boards and aprons were provided
Cunningham-Sabo et al. [38] (2014)	none described	none described	bilingual curriculum, affordable ingredients; focus on diverse cultural traditions
D’Adamo et al. [39] (2016)	none described	students, teachers, community-based health professionals involved in curriculum development	spices selected based on accessibility, cultural acceptability, affordability, palatability, versatility, health benefits, familiarity, novelty
Davis et al. [40] (2016)	Social Cognitive Theory and Self Determination Theory	pilot tested with 4th and 5th grade students; tested again in cluster RCT with predominantly low-income Hispanic 3rd–5th grade students	lessons were culturally tailored
Gatto et al. [41] (2017)	self-efficacy	pilot tested with predominantly low-income Hispanic students prior to finalizing program	none described although developed for urban Latino upper elementary aged children
Jarpe-Ratner et al. [42] (2016)	none described	none described	recipes designed to be affordable, flexible, and consistent with dietary guidelines (2010)
Liquori et al. [43] (1998)	Social Cognitive Theory	pilot tested classroom and lunchroom components—adjusted based on results and feedback	pilot tested classroom and lunchroom components—adjusted based on results and feedback
Overcash et al. [44] (2018)	Social Cognitive Theory	none described	designed for low-income families (no information provided on how this was accomplished)

**Table 3:** Intervention characteristics.

Author (Year)	Components	Primary Intervention Focus	Delivery Mode	Parent Involve-ment *	# of Sessions	Session Length	Program Duration	Program Leader(s)	Leader Training	Delivery Location(s)
Bell et al. [36] (2018)	program focus—nutrition education, cooking, gardening; Game: cooking and gardening; classroom curriculum: nutrition education; cooking demonstrations; practice; reflection; family home activities—materials provided	child	game (played in class on tablet), in-class lessons, in-home activities	+++	3 game sessions, 3 class lessons, 3 in-home activities	Games and lessons were each an hour long, and in-home activities spanned the course of 3 days per week	3 weeks	game (independent); teacher (classroom); home (family)	Teachers were trained	Games played and lesson taught in classroom. The in-home activities were at home
Chen et al. [37] (2014)	Recipe demonstrations, recipe card info lessons, tasting activities. Family food kits were given to students to take home (cooking equipment, spices). Backpack of equipment also provided	both	classroom, home	+++	1 session per month (1–2 recipes)	20 min to present in-class activities for one recipe	Feb–May	nutrition educator and teacher	none described	classroom and home
Cunningham-Sabo et al. [38] (2014)	cooking and/or tasting sessions	child	hands-on cooking classes and/or tasting sessions in classroom; classroom meals served in school cafeteria several times a month	+	1 introductory session; 5 cooking and/or FV tasting sessions	1 h introductory session; 2 h cooking sessions; 1 h tasting lessons	school year	Parents invited to volunteer. FV tastings led by classroom teachers. Cooking lessons led by Cooking with Kids food educators	none described	classroom; school cafeteria
D’Adamo et al. [39] (2016)	Spice MyPlate intervention was 6 weekly nutrition education sessions focused on using spices and herbs in a diet + a 1 h grocery tour + 2 h of cooking sessions	child	classroom lessons (health class), grocery tour, cooking sessions	−	1 h standard nutrition education, 6 sessions of My Plate curriculum, 1 grocery tour, 2 h of cooking sessions	nutrition lessons were 1 h long, grocery tour was 1 h, and there was a total of 2 h of cooking sessions	6 weeks	Chefs led the cooking sessions; Health Corps coordinator led the nutrition lessons	none mentioned	school (health class)
Davis et al. [40] (2016)	gardening, cooking, nutrition	child	hands-on, instructional	−	12	90 min	12 weeks	nutrition and garden educators with strong backgrounds in cooking, nutrition, gardening	none described	school (after-school program)
Gatto et al. [41] (2017)	gardening, cooking, nutrition	child	hands-on, demonstration	+++ (parallel program for parents)	12	90 min	12 weeks	educators with nutrition or gardening backgrounds	none described	school (school garden)
Jarpe-Ratner et al. [42] (2016)	nutrition education, culinary skills, and meal preparation, meal sharing, and discussion	child	hands-on, instructional	+	10 per semester	30-min lectures, 75-min instruction on culinary skills and prep, 15-min of meal sharing, conversation	10 weeks in a school semester	chef-instructors	chef-instructors went through 2 h training by Common Threads staff	school (after-school program)
Liquori et al. [43] (1998)	school lunch component; classroom component (cooking and tasting sessions OR participatory activities without cooking and tasting); parent and community component	child	hands-on, instructional	+++	−	60-90 min for cook shop; 45 min for food and environment lessons	school year	food service staff led cafeteria component; classroom teachers, parents, and college students were Cook Shop instructors	Cook Shop instructors had two 3-h training sessions; food service staff had one 3 h training session; program staff met with parent assistants and volunteer college students before and after each session for training support	school
Overcash et al. [44] (2018)	demonstration, food preparation, nutrition education lessons, and a meal. Families were given a bag of groceries needed to prepare the meal at home	both	hands-on, instructional	+++	6	2 h	September 2014–June 2016	chefs, nutrition educators	chefs and nutrition educators went through training sessions	11 different host sites

* Legend: (−) none; (+) minimal—i.e., recipes, newsletters; (++) modest—volunteer; (+++) major—home component with parent involvement.
